# Oxidative Stress and Histopathological Changes in Gills and Kidneys of *Cyprinus carpio* following Exposure to Benzethonium Chloride, a Cationic Surfactant

**DOI:** 10.3390/toxics10050227

**Published:** 2022-04-29

**Authors:** Stefania Gheorghe, Miruna S. Stan, Daniel N. Mitroi, Andrea C. Staicu, Marius Cicirma, Irina E. Lucaciu, Mihai Nita-Lazar, Anca Dinischiotu

**Affiliations:** 1 National Research and Development Institute for Industrial Ecology (ECOIND), 71–73 Drumul Podu Dambovitei, 060652 Bucharest, Romania; stefania.gheorghe@incdecoind.ro (S.G.); irina.lucaciu@incdecoind.ro (I.E.L.); mihai.nita@incdecoind.ro (M.N.-L.); 2 Department of Biochemistry and Molecular Biology, Faculty of Biology, University of Bucharest, 91–95 Spl. Independentei, 050095 Bucharest, Romania; daniel.mitroi@abbvie.com (D.N.M.); cristina.staicu@bio.unibuc.ro (A.C.S.); mariuscicirma@gmail.com (M.C.); adin@bio.unibuc.ro (A.D.); 3 Research Institute of the University of Bucharest (ICUB), University of Bucharest, 050657 Bucharest, Romania; 4 AbbVie Inc., 2525 DuPont Dr, Irvine, CA 92612, USA

**Keywords:** ecotoxicology, benzethonium chloride, cationic surfactant, fish, gill, kidney

## Abstract

One cationic surfactant with a wide spectrum of microbiocidal activity is benzethonium chloride (BEC). Despite being widely used, the toxicity data on vertebrate organisms are limited. Therefore, we aimed to evaluate within this study the acute toxicity of BEC on the gills and kidneys of *Cyprinus carpio* (European carp). An alteration of the antioxidant enzymes activities (glutathione reductase, glutathione peroxidase and glutathione S-transferase) was noticed after 96 h of exposure, along with an elevation of lipid peroxidation and decreased concentration of reduced glutathione, which confirmed that BEC was able to induce toxicity to these tissues. These metabolic effects were correlated with unspecific structural changes observed in gills and kidneys, having moderate degree of severity (such as an increase of melanomacrophages aggregation incidence and cytoplasm vacuolation of goblet cells in collecting tubules) and generally being compatible with life for the exposure time studied. The most severe structural effects were observed in gills after 96 h, noticing a lamellar aneurysm, hemorrhages and lamellar epithelium disruption due to the blood vessels and pillar cells damages and increased blood flow inside the lamellae. By our research we can confirm the utility of biochemical and histological analyses in the fish organs as tools for monitoring the water quality and ecotoxicological potential of chemicals.

## 1. Introduction

The aquatic systems are the target of chemical pollution, the surfactants compounds being the most common pollutants that result from domestic and industrial activities. Surfactants are a group of chemical compounds containing a polar head group and a nonpolar hydrocarbon tail, used for cleaning applications [1]. Previously, it was shown that ~60% of surfactant products enter the aquatic environment [2]. The international regulations set limits only for two classes of surfactants (e.g., anionic and nonionic), while the cationic and amphoteric surfactants remain uncovered by legislation in many countries, although many cosmetics, detergents or biocide products frequently contain this kind of compounds. Therefore, the environmental studies from the last decades have been more focused on anionic and nonionic surfactants [3,4,5,6], while for cationic surfactants the data are limited.

The surfactants’ production is increasing and it is considered that it will reach more than 60 billion of dollars by 2025 [7,8]. This estimation is based on the high demand for products containing nonionic and cationic surfactants with biocide activity due to the COVID-19 pandemic situation. The cationic surfactants from the quaternary ammonium compounds class used in disinfectant products actually became one of the most efficient products for SARS-CoV-2 virus inactivation on surfaces [9].

In surface waters, the cationic surfactants were detected from 0.01 to 3900 µg/L [10,11]. Predicted environmental concentrations (PEC) were estimated from 0.002 to 0.2 mg/L [12], the level for the Danube River being 10 µg/L [13]. Their toxic effects on aquatic organisms were estimated to be exerted by environmental concentrations between 0.07 and 42 mg/L, based on trophic levels and other abiotic and biotic factors [14]. Benzethonium chloride (BEC), known also as Hyamine 1622, is a quaternary ammonium salt from the group II of nonhalogenated benzyl substituted quats [15], with cationic properties currently used in detergents (0.1–1.6%), sanitation products (>50%) and cosmetics (<1%) [16]. Their structure has at least one hydrophobic hydrocarbon chain linked to a positively charged nitrogen atom, and the other alkyl groups can be short-chain substituents (methyl or benzyl groups) [17]. BEC has a wide spectrum of microbiocidal activity, being effective against various types of bacteria, fungi and viruses [18]. Due to these properties, the use of BEC is strictly regulated by international legislations [19]. The risk coefficient of BEC in the aquatic medium was predicted to be higher than 1, especially for algae growth and *Daphnia magna*, at concentrations below 10 μg/L. BEC is classified as “acute toxic, class 1 of toxicity” [19] with severe effects on crustaceans, algae and bacteria [13].

According to Bindu et al. (2001) [20], the cationic surfactants induce different antioxidant and metabolic profiles in vivo, being the most toxic compared to nonionic, anionic and amphoteric surfactants [9]. Cationic surfactants are able to generate superoxide and hydrogen peroxide that can induce oxidative stress, disturbing the normal function of organs [21]. Previously, it was shown that BEC has the ability to induce unspecific structural changes in fish liver [22]. Generally, the surfactants affect in the first step the gills and epidermis of aquatic vertebrates because of their direct contact with the polluted aquatic medium through wide contact surfaces and their thin epithelium [23]. These have the ability to pass through the cell membrane of aquatic organisms [24], exerting their toxic effects through the interaction with lipid membranes, which could result in membrane integrity loss and ion permeability increasing [25]. Therefore, metabolic processes could be affected in the body cells by the oxidative stress induced in the presence of high doses of surfactants [26].

The toxicity data available on cationic surfactants are limited, most of the studies being focused on the biocidal efficiency on invertebrate organisms. Therefore, we aimed to evaluate within this study the acute toxicity of BEC on the gills and kidneys of *Cyprinus carpio*. A number of characteristic enzymes for oxidative stress, such as superoxide dismutase (SOD), catalase (CAT), glutathione-peroxidase (GPx), glutathione S-transferase (GST), glutathione reductase (GR), glucose-6-phosphate dehydrogenase (G6PDH), and lactate dehydrogenase (LDH) were analyzed. Lipid peroxidation and reduced glutathione (GSH) levels were also measured. In addition, in order to evaluate the structural injuries of organ tissues, histopathological changes were investigated.

## 2. Materials and Methods

### 2.1. Chemicals

The cationic surfactant tested was benzethonium chloride (BEC) (IUPAC name: benzyl-dimethyl-[2-[2-[4-(2,4,4-trimethylpentan-2-yl) phenoxy] ethoxy] ethyl] azanium chloride; commercial name: Hyamine 1622). It was purchased from Fluka Chemie GmbH (Buchs, Switzerland), having a purity higher than 96%, CAS no. 121-54-0 and molecular weight of 448.08 g/mol. Chemical reagents for surfactant detection (chloroform, disulfide blue VN 150, ethanol, 1 N sulfuric acid), and biochemical and histological reagents were obtained from Sigma-Aldrich (St. Louis, MO, USA), having a purity higher than 98%.

### 2.2. Fish

*Cyprinus carpio* is a freshwater fish common in surface waters of Europe, being considered a very good model organism for environmental studies [27] and it was used within our investigation. One-year-old carp with health and origin certificate no. 54031 were collected from an unpolluted fish farm in Nucet (Romania). Fish were acclimatized in laboratory conditions within the National Research and Development Institute for Industrial Ecology (ECOIND, Bucharest, Romania). In vivo studies were performed in conformity with the Guide for the Use and Care of Laboratory Animals [28] and Organization for Economic Co-operation and Development (OECD) recommendations regarding reduction of animal suffering and number of animals sacrificed [29]. The research was authorized and carefully supervised by the Commission of Ethics and Professional Deontology of ECOIND (Intern Regulation no. 18671).

### 2.3. Sub-Lethal Toxicity Experiment

A total of 40 fish (length 15 ± 2 cm, weight 58 ± 10 g) were randomly distributed in 100 aquaria and exposed to 1 mg/L BEC (nominal concentration) solution for a period of 24, 48, and 96 h in order to perform an acute semi-static toxicity test in accordance with the adapted OECD test no. 203 [30].

A solution of 1 mg/L BEC was prepared with free chlorine tap water and put in test aquaria, 80% being renewed every 24 h. Another 40 fish were considered the control group as they were not incubated with BEC solution. Fish did not receive food during the whole period of incubation and were maintained under a natural photoperiod of 12 h light/12 h dark. After 24, 48, and 96 h, 5 fish from the BEC group and 5 fish from the control group were sacrificed. The average length of the fish was 13.38 ± 1.98 cm (control group) and 16.2 ± 1.63 cm (BEC group), and the average body mass was about 59 ± 11.40 g (control group) and 65.4 ± 10.25 (BEC group) [22]. Gills and kidneys were collected and kept at −80 °C for biochemical tests. Fragments of these organs were also taken, fixed in 10% buffered formalin and processed for examination by light microscopy.

The concentration of BEC used within our research was selected based on previous studies regarding the assessment of lethal concentration 50 (LC50) for the same fish species [14]. The equation of Sprague was used to predict the maximum acceptable toxicant concentration (MATC): MATC = LC50 value after 96 h (which was 4.57 mg/L) × 0.1. The selected concentration (nominal 1 mg/L BEC, analytic 0.78 ± 0.15 mg/L) used for the fish acute exposure caused no mortality in the exposure period. Using concentrations between 3 and 40 mg/L, we registered 40% to 100% mortalities (data not showed).

Therefore, to achieve a minimum concentration of 0.5 mg/L in the test aquaria in the conditions of surfactant hydrophobic properties, a nominal concentration of 1 mg/L BEC was chosen to prepare the stock solution. Based on our research performed on the local municipal wastewater and surface water, the presence of this cationic surfactant was in the range of 0.003–0.35 mg/L. However, the total concentration of anionic and nonionic surfactants is restricted to 0.5 mg/L for the wastewater discharged in water bodies [14].

### 2.4. Analytical Control of BEC

The analytical control of cationic surfactant (BEC) was performed daily, before and after changing the solution. The cationic surfactant concentration was determined colorimetrically as its disulphine blue ion-association compound, extractable into chloroform, based on the German standard method—DIN 3849/20:1989 (as specified on Annex IIC of Detergent Regulation no. 648/2004). This method was chosen based on the disulphine blue VN 150 high sensitivity, the wavelength of maximum absorption for the disulphine blue–cationic surfactant complex was at 628 nm in chloroform, using a UV–vis spectrometer (Specord 205, Analytik Jena, Germany). The calibration curve was performed for six BEC concentrations and a good linear relationship between absorbance and concentration was obtained over the range of 0.1–4 mg/L. The method was verified for linearity, accuracy and precision. The correlation coefficient R^2^ was 0.9904, the percent recovery (*n* = 3) was 98.05, the percent relative standard deviation (RSD) (*n* = 3) was 0.222, the detection limit was 0.003 mg/L, the quantification limit was 0.035 mg/L and the extended measurement uncertanity was 10.33%.

### 2.5. Biochemical Investigations

#### 2.5.1. Obtaining the Tissue Homogenate

Gills and kidney tissues (1/10 weight/volume) were homogenized with an ice-cold buffer (pH 7.4) containing 0.1 M Tris and 5 mM ethylenediaminetetraacetic acid (EDTA) for two cycles of 2 min at 20 movements per second with a mixer mill (type MM301, Retsch GmbH & Co, Haan, Germany). Homogenates were incubated for 60 min at 4 °C and centrifuged at 10,000× *g* for 30 min at 4 °C. Supernatants were collected and protein content was spectrophotometrically determined at 660 nm based on Lowry’s method with a standard of bovine serum albumin [31].

#### 2.5.2. Measurement of Enzyme Activities

Catalase (CAT) activity was determined by recording the decrease of H_2_O_2_ concentration at 240 nm based on the protocol described by Aebi in 1984 [32]. Superoxide dismutase (SOD) was measured based on NADH oxidation evidenced by the decrease in absorbance at 340 nm using a Tecan Genios microplate reader (Tecan, Salzburg, Austria) [33]. Glutathione peroxidase (GPx) activity was estimated with tert-butyl hydroperoxide and GSH as substrates [34]. The conversion of NADPH to NADP^+^ due to the reduction of GSSG to GSH in the reaction catalyzed by glutathione reductase (GR) was followed by recording the changes in absorption intensity at 340 nm. GR activity was evaluated using the Golberg and Spooner’s method [35], which involves a mix of 0.1 M phosphate buffer (pH 7.4) with 0.66 mM of oxidized glutathione (GSSG) and 0.1 mM NADPH. Activity of glutathione S-transferase (GST) was assessed by evaluating the rate of 1-chloro-2, 4-dinitrobenzene (CDNB) conjugation with GSH according to Habig [36]. Glucose 6-phosphate dehydrogenase (G6PDH) activity was evaluated based on the speed of NADPH formation, which appeared as an increase in absorbance value at 340 nm. Lactate dehydrogenase (LDH) activity was measured based on the speed of absorbance decrease at 340 nm during 5 min due to NADH oxidation.

The enzyme activities were divided by protein concentration to express them in terms of units of activity per milligram of protein (U/mg) and as a percentage from control.

#### 2.5.3. Measurement of GSH Level

GSH level was measured with the Glutathione Assay Kit (Sigma-Aldrich, St. Louis, MO, USA) following the manufacturer’s instructions. The absorbance of the mix solution was measured at 405 nm with a Tecan Genios microplate reader (Tecan, Salzburg, Austria).

#### 2.5.4. Measurement of Lipid Peroxidation

Lipid peroxidation in gills and kidneys was estimated by measuring malondialdehyde (MDA) concentration based on a fluorometer [37]. The samples were incubated with 0.1 M HCl for 20 min at room temperature. Then, 0.025 M thiobarbituric acid was added, and the fluorescence intensity was recorded after 65 min of incubation at 37 °C, using the 520/549 nm (excitation/emission) wavelengths on a FP-6300 spectrofluorometer (JASCO, Tokyo, Japan). Different concentrations of 1,1,3,3-tetramethoxypropane in the range of 0.05–0.5 µM were used to obtain a calibration curve and the MDA amount in the samples.

#### 2.5.5. Statistical Analysis

The biochemical tests were run in three technical replicates for each fish (*n* = 5). The comparison between control group and BEC group was assessed by a one-way ANOVA followed by a post hoc Bonferroni test within GraphPad Prism software (version 9.0.0; GraphPad Software, Inc., La Jolla, CA, USA). A *p* value lower than 0.05 was considered statistically significant.

### 2.6. Histological Staining

Histological modifications produced by the exposure of *Cyprinus carpio* to 1 mg/L BEC were analyzed by hematoxylin and eosin staining. Gill and kidney tissues were collected after 24, 48, and 96 h of incubation and maintained in 10% formalin for 24 h to avoid the tissues’ degradation. The dehydrating and tissue clarification steps were performed using consecutive incubations with ethanol of increasing concentrations (70°, 90° and 100°) and toluene. The samples were embedded in paraffin wax at 60 ℃ and sectioned with a Lipshaw rotary microtome set at a size of 5–6 µm. These sections were put on slides and kept overnight at 37 ℃. Further, the slides were deparaffinized using toluene and washed with ethanol of decreasing concentrations (100°, 90° and 70°). Next, a staining with hematoxylin and eosin was performed, followed by a dehydration in ethanol and clarification with toluene. Canada balsam was applied, and the sections were visualized on an Olympus BX43 light microscope (Olympus, Tokyo, Japan). The histopathological changes within each type of tissue were analyzed in ten randomly selected sections obtained for each fish.

## 3. Results

### 3.1. Characterization of Water Parameters

The average analytical concentration of BEC measured during the toxicity experiment was 0.78 ± 0.15 mg/L (variation coefficient of 19.5%). The variation of BEC concentration was maintained below the limit of 20%; therefore, the value of 0.78 ± 0.15 mg/L could be used for the toxicity assay.

The abiotic parameters were monitored during the experiment: pH 7.4 ± 0.1, ambient temperature 20 ± 2 °C, dissolved molecular oxygen 6 ± 1 mg O_2_/L, total hardness as CaCO_3_ 184 ± 7.2 mg/L, solid matter 8.8 ± 4.3 mg/L, chemical oxygen demand 20.2 ± 5.92 mg/L. After 96 h of incubation, all fish were alive in both groups. The control animals satisfied the validity of test requirements, having a mortality less than 10%. In addition, no visible behavior changes were noticed.

### 3.2. Effects Induced by BEC on Cellular Antioxidant System

**Figure 1:**
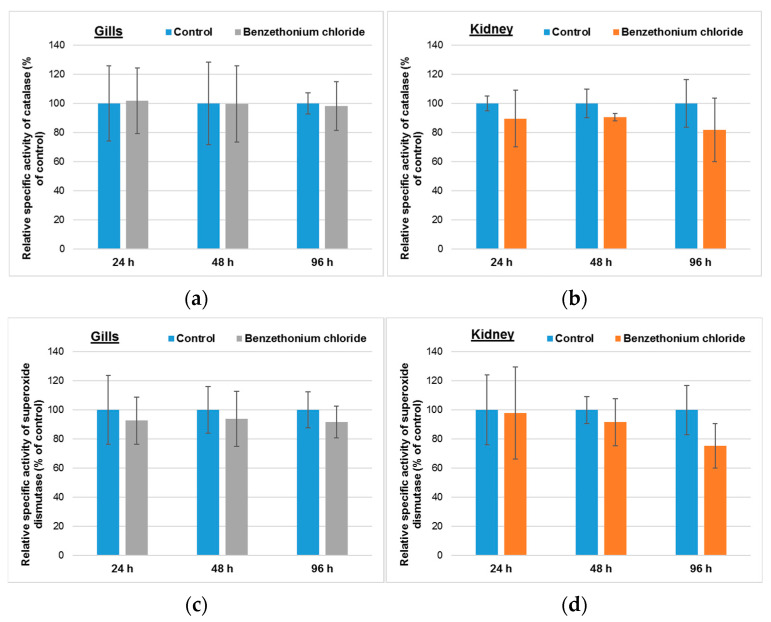
Catalase (**a**,**b**) and superoxide dismutase (**c**,**d**) activities in gills (**a**,**c**) and kidney (**b**,**d**) of *Cyprinus carpio* incubated with 1 mg/L of BEC (analytical concentration of 0.78 ± 0.15 mg/L) for up to 96 h. Data are presented as mean levels ± standard deviation (*n* = 5) and expressed relative to control.

As it can be seen in Figure 1, the fish exposure for up to 96 h to 1 mg/mL BEC (the analytical concentration was 0.78 ± 0.15 mg/L) did not induce any significant changes on the specific activities of CAT and SOD in gills. Regarding the effect on kidney, a decrease by 18% and 25% from the control level for CAT and SOD activity, respectively, was noticed after 96 h.

**Figure 2:**
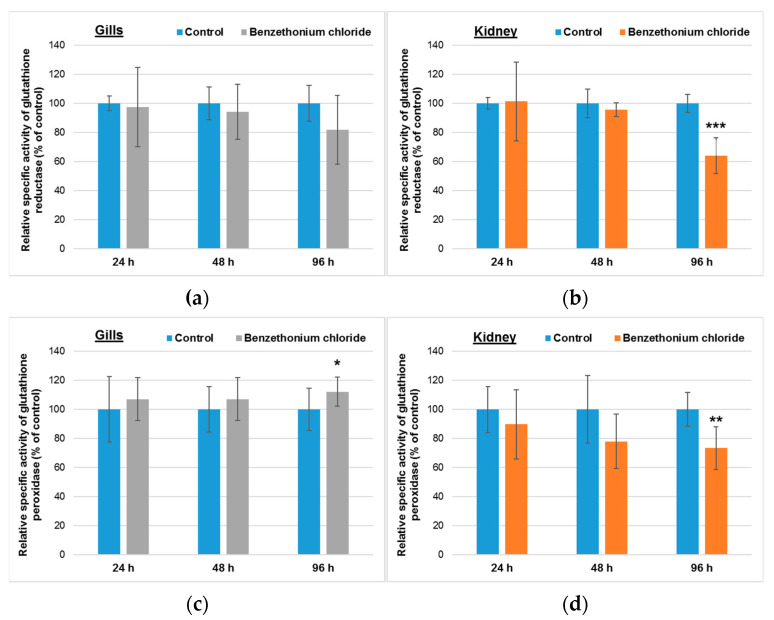

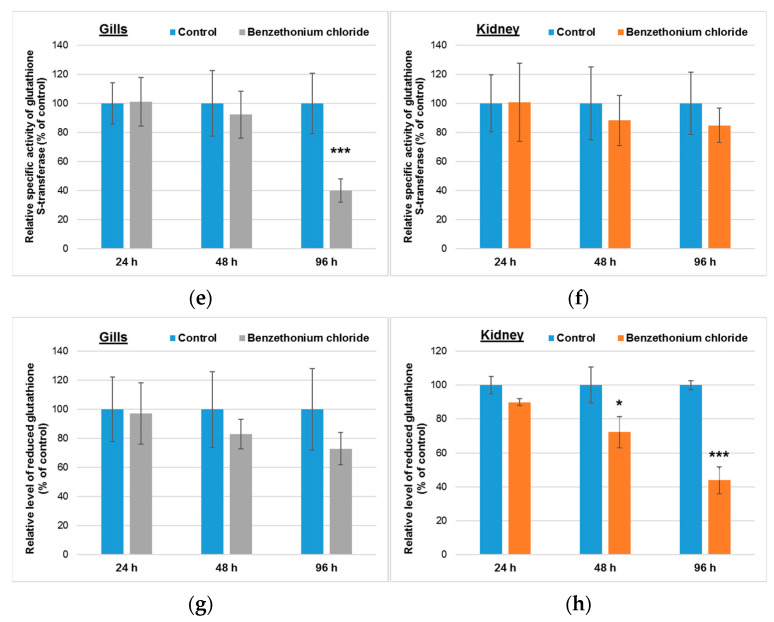
Activities of glutathione reductase (**a**,**b**), glutathione peroxidase (**c**,**d**), glutathione S-transferase (**e**,**f**) and concentration of GSH (**g**,**h**) in gills (**a**,**c**,**e**,**f**) and kidney (**b**,**d**,**f**,**h**) of *Cyprinus carpio* incubated with 1 mg/L of BEC (analytical concentration of 0.78 ± 0.15 mg/L) for up to 96 h. Data are presented as mean levels ± standard deviation (*n* = 5) and expressed relative to control. * *p* ≤ 0.05, ** *p* ≤ 0.01 and *** *p* ≤ 0.001 compared to control.

A nonsignificant diminution of GR activity was registered in gills after the longest period of incubation (Figure 2a), but a significant increase by 12% compared to control was induced for GPx specific activity (Figure 2c). The important decrease of GST activity (Figure 2e) by 60% of the control level after 96 h could be correlated with the time-dependent decrease of GSH concentration in the gill tissue (Figure 2g).

In kidney, a significant decrease of GR specific activity by 36% relative to control was observed after 96 h (Figure 2b). In addition, the activity of GPx diminished by 27% compared to control after 96 h, although no great differences were recorded after 24 h exposure (Figure 2d). Whereas the GST specific activity (Figure 2f) was not modified significantly during the whole exposure interval, the GSH level decreased significantly in a time-dependent manner, by 28% and 56%, after 48 h and 96 h, respectively, compared to the control level (Figure 2h).

### 3.3. Effects Induced by BEC on Enzymes-Generating Reducing Equivalents

**Figure 3:**
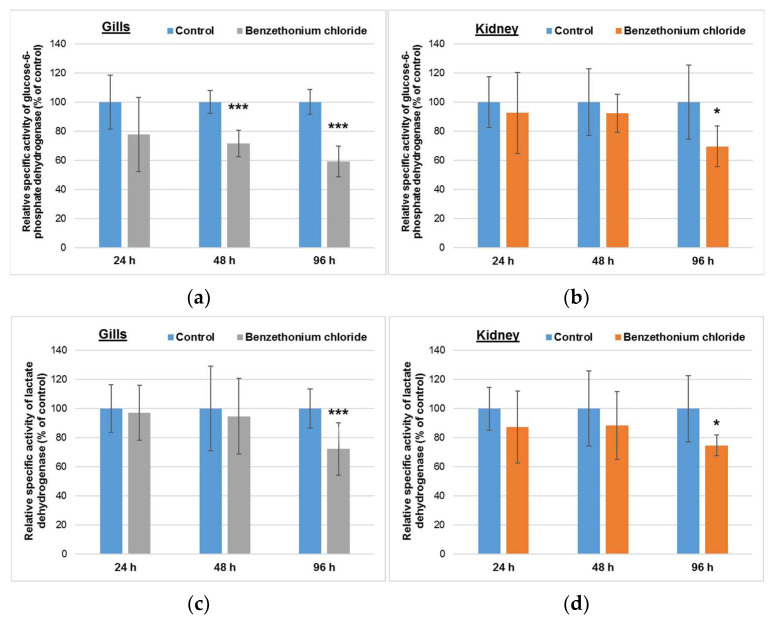
Glucose-6-phosphate dehydrogenase (**a**,**b**) and lactate dehydrogenase (**c**,**d**) activities in gills (**a**,**c**) and kidney (**b**,**d**) of *Cyprinus carpio* incubated with 1 mg/L of BEC (analytical concentration of 0.78 ± 0.15 mg/L) for up to 96 h. Data are presented as mean levels ± standard deviation (*n* = 5) and expressed relative to control. * *p* ≤ 0.05 and *** *p* ≤ 0.001 compared to control.

Analyzing Figure 3, it can be seen that in gills, the G6PDH specific activity decreased significantly compared to control in a time-dependent manner, by 28% and 41% after 48 h and 96 h, respectively, whereas the LDH activity decreased significantly by 28% from the control level after 96 h. In contrast, the G6PDH specific activity in kidney was diminished significantly by 30% only after 96 h, the levels being near that those of the control group for 24 h or 48 h exposure. Moreover, in the case of this organ, the same decrease pattern was registered for LDH specific activity.

### 3.4. Effects Induced by BEC on Lipid Peroxidation

**Figure 4:**
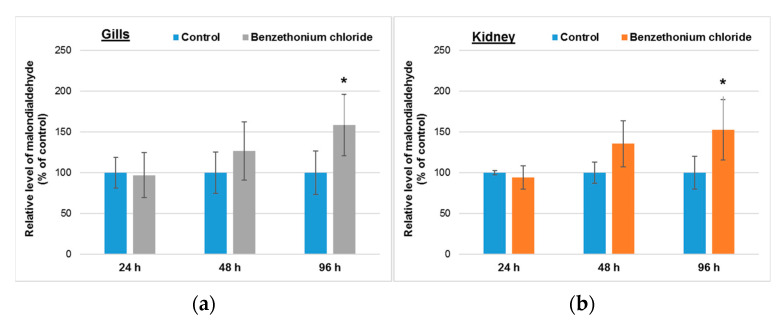
Lipid peroxidation level in gills (**a**) and kidney (**b**) of *Cyprinus carpio* incubated with 1 mg/L (analytical concentration of 0.78 ± 0.15 mg/L) of BEC for up to 96 h. Data are presented as mean levels ± standard deviation (*n* = 5) and expressed relative to control. * *p* ≤ 0.05 compared to control.

Malondialdehyde represents a marker of lipid peroxidation, and its generation can be induced by toxic xenobiotics. Figure 4 shows that its level raised in a time-dependent way in the gills and kidneys of *Cyprinus carpio* incubated with 1 mg/L of BEC (analytical concentration of 0.78 ± 0.15 mg/L).

### 3.5. Histopathological Changes Induced by BEC

Gills and kidneys represent targets of histopathological changes induced by environmental contamination [7]. Within our study, changes in tissue structure of fish gills and kidneys were observed and qualitatively described in accordance with the lesions presented previously by [38,39], respectively.

**Figure 5:**
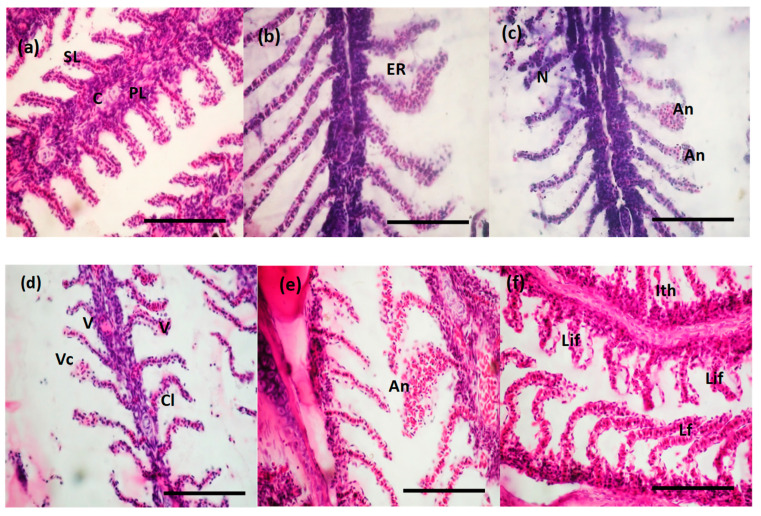
Microphotographs of hematoxylin and eosin staining to reveal the gill histopathological changes observed after BEC exposure. Gill sections of (**a**) control group: primary lamella (PL), secondary lamella (SL), cartilaginous support (C), gill filaments showing no histological alteration; (**b**,**c**) fish incubated with BEC for 24 h: epithelial rupture (ER), aneurysm or telangiectasia (An), erosion of the secondary lamellae (necrosis) (N); (**d**) fish incubated with BEC for 48 h: vasodilatation (V) and vascular congestion (Vc), curling of the secondary lamellae (Cl); (**e**,**f**) fish incubated with BEC for 96 h: aneurysm (An) with different levels of severity, lifting of lamellar epithelium (Lif), lamellar fusion (Lf), inter-lamellar tissue hyperplasia (Ith). Scale bars correspond to 100 µm.

A normal structure with primary and secondary lamellae and a cartilaginous core was evidenced for the gill tissue of the control fish (Figure 5a). After the first 24 h of exposure to the surfactant, several modifications in the morphology of branchial lamellae were visualized. The most frequently observed effects presented a low level of severity, compatible with a normal activity of the gills: marginal channel dilatation, lamellar epithelium lifting, epithelial hyperplasia and very rarely the fusion of secondary lamellae (Figure 5b). These abnormalities are associated with the defense mechanism that generates a distance between the external environment and the blood and a barrier against the possible infiltration of the surfactant. Among the most severe damages that put in danger gill functions, we reported a lamellar necrosis and aneurysm and hemorrhages along with a lamellar epithelium fissure due to the blood vessels and pillar cells lesions and to increased blood flow inside the lamellae (Figure 5d–f). Vacuolization curling of secondary lamellae and the aneurism were more prominent after 96 h. The observed injuries are considered unspecific and can be triggered by different water pollutants.

**Figure 6:**
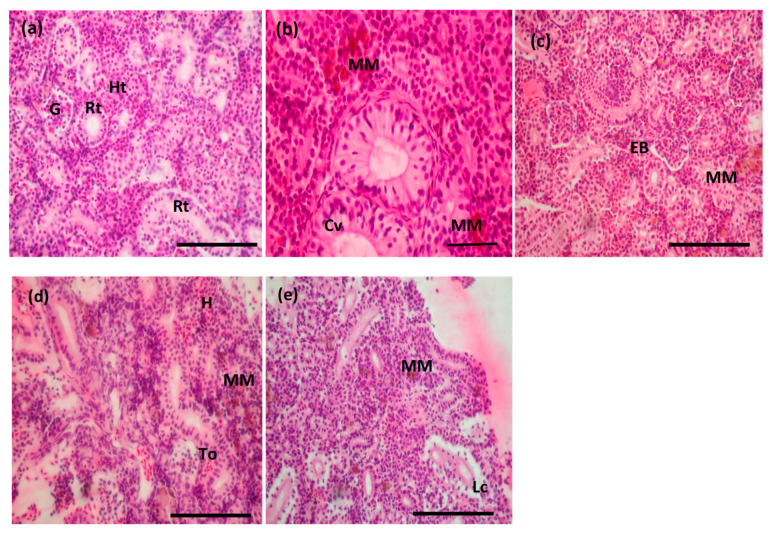
Microphotographs of hematoxylin and eosin staining to reveal the kidney histopathological changes observed after BEC exposure. Kidney sections of (**a**) control group: kidney tissues showing normal renal feature with many renal tubules (Rt), glomeruli (G) and hematopoietic tissue (Ht); (**b**–**e**) fish incubated with BEC for 96 h: contraction of glomerulus and expansion of Bowman space (EB), loss of cellular integrity (Lc), melanomacrophages aggregation (MM), cytoplasm vacuolation of goblet cells (Cv), hemorrhagic area (H), tubular occlusion (To). Scale bars correspond to 100 µm (**a**,**c**,**d**,**e**) and 50 µm (**b**).

The excretory trunk region of the kidney was examined in control samples highlighting the segments of the nephrons, which have specific structure and functions: renal corpuscle, consisting of the glomerulus included in the Bowman’s capsule, proximal and distal convoluted tubules and collecting tubules, supported by hematopoietic tissue (Figure 6a). The following circulatory, regressive and inflammatory changes appeared due to the 96 h exposure to BEC: hemorrhagic areas near renal tubules, increase of melanomacrophages aggregation, expansion of Bowman space, cytoplasm vacuolation of goblet cells lining the collecting tubules, degeneration of the tubular epithelium and renal tubules occlusion during epithelial cells proliferation (Figure 6b–e).

## 4. Discussion

Fish gills are in permanent contact with water, being the primary target of pollutants [40], and are bioconcentrating cationic surfactants, because these adhere strongly to cell membranes [41]. Further, the kidney receives the largest amount of branchial blood, and the lesions identified are expected to be good markers for environmental pollution [42]. Therefore, we selected these two organs for evaluating the consequences of *Cyprinus carpio* exposure to one cationic surfactant, BEC.

Cationic surfactants interact by ionic bonds with negatively charged humic acids and clay colloids and are accumulated in sediments. However, a dissolved fraction remains in water and can be uptaken by aquatic organisms [43]. Their microbicidal activity is exerted by the destabilization of cell membrane [44], especially at concentrations close to their critical micellar concentrations [45].

Taking into consideration that BEC concentration in the aquarium during the exposure was low, and most probably an important part of it was absorbed by mucus, BEC was uptaken in low amount in gills and its contribution to the depolarization of mitochondrial inner membrane could have been low enough to generate an important level of superoxide anion. As a result, in the absence of a higher quantity of substrate, Mn-SOD specific activity did not significantly change after up to 96 h of exposure (Figure 1). Furthermore, a brachial elimination of this xenobiotic could also occur [46], diminishing its concentration in this organ.

Due to its hydrophobic tail, BEC probably diffuses through cell membranes and is distributed through the circulatory system. At this level, it could interact with albumin, the most important plasmatic protein by hydrophobic and ionic bonds, and later on, it might be released to different tissues, metabolized and excreted. In the kidney, due to the glomerular filtration and concentration of urine, the level of BEC and its metabolites could increase. As a result, most probably, a mitochondrial disfunction was developed, and an important quantity of superoxide was formed. However, at high levels of superoxide, eukaryotic Mn-SOD becomes less efficient, generating lower hydrogen peroxide levels [47]. These could explain the nonsignificant decrease of total SOD and CAT specific activities in kidneys within our experiment (Figure 1).

In the presence of hydrogen peroxide, superoxide generates hydroxyl radical according to the Haber–Weiss reaction. Hydroxyl radicals are extremely reactive and attack immediately the polyunsaturated fatty acids, generating lipid peroxidation, whose biomarker is malondialdehyde. This is a cascading process that amplifies over time.

In both analyzed organs, the levels of malondialdehyde increased in a time-dependent manner, being significantly higher after 96 h compared to 24 h of exposure (Figure 4). This suggests the installation of an oxidative stress, that represents an imbalance between pro-oxidants and antioxidants, favored by the presence of some xenobiotics or physical factors.

In gills, malondialdehyde was probably transformed in the presence of GSH within the reaction catalyzed by GPx, because a significant raise of this specific activity was observed after 96 h (Figure 2). The low activity of GST after the same period of time could suggest that BEC, an electrophilic compound, was not directly conjugated with GSH. Probably, the use of GSH in the reaction catalyzed by GPx diminished the total concentration of GSH, taking in account that this could not be regenerated from GSSG in the reaction catalyzed by GR. The decrease of the GR specific activity in a time-dependent manner might be correlated with that of G6PDH (which has the same pattern), due to the fact that the reaction catalyzed by the latter enzyme is involved in NADPH generation, a cofactor essential for the activity of GR.

In contrast to gills, malondialdehyde was not decomposed in the reaction catalyzed by GPx in kidneys, because the activity of this enzyme decreased significantly after 96 h of exposure. The level of GSH decreased significantly in a time-dependent manner and was correlated with the low GR and G6PDH specific activities (Figure 2). GSH depletion indicated the accumulation of free radicals in cells, being also correlated with the decrease of GST activity in gills and the high level of MDA after 96 h. Comparable results were shown in the carp liver exposed to BEC [22]. Previously, it was shown that other cationic surfactants, such as cetyltrimethylammonium bromide and cetylpyridinium chloride, can induce at sublethal concentrations severe oxidative stress and lipid peroxidation in the liver, gill, kidney and heart of freshwater species [48,49].

Comparing the relative concentrations of GSH in the gills and kidney of fish incubated with BEC, we noticed that after 96 h of exposure, the GSH concentration in kidney was lower by ~20% compared to the one recorded in gills, suggesting a higher oxidative stress level.

The decreased activities of G6PDH in gills and kidneys (Figure 3) could be explained by the possible effect of BEC on glucose uptake [50]. LDH catalyzes the reversible reaction between pyruvate and lactate, during anaerobic glycolysis. BEC uptaken by the gills’ and kidneys’ cells probably interacted electrostatically with pyruvate, and after 96 h, the decrease of its concentration was low enough to decrease LDH specific activity.

In our study, the fish exposure to BEC showed gradual structural changes in the histology of both studied organs (Figure 5 and Figure 6), which were identified also for other types of toxicants [38,51,52]. The observed structural changes within the gills can be classified in two categories. The first one included the changes with reduced severity, considered as defense modifications, and the second one with severe changes occurred as a results of cationic surfactant toxicity. Inflammatory lesions, such as dilatation of marginal channel, lifting of the lamellar epithelium, epithelial hyperplasia and fusion of the secondary lamellae, were observed especially after 24 and 48 h of BEC exposure, being considered as a defense mechanism against pollutant on the gill surface. The mucus accumulation and the hyperplasia caused the lamellar fusion, which was needed in order to increase the area of exchange between blood and available oxygen [53,54]. The most severe effects were observed after 96 h of exposure, when lamellar aneurysm (Figure 5c) and hemorrhages were noticed, and also lamellar epithelium disruption due to the blood vessels and pillar cells damage and increased blood flow inside the lamellae (Figure 5d–f). These changes are noted as unspecific because various chemicals are able to produce them. Comparable results obtained for *Rita rita* (Asian fish species) intoxicated with anionic surfactant showed a gradual decrease in lipid fractions in the epithelial cells of the gills [55] which can lead to a loss of respiration and osmoregulation. Cationic surfactants, such as alkyl dimethyl benzyl ammonium chloride, in a concentration of 3 ppm, caused severe damage to the gills by lamella fusion, necrotic damages, membrane vesicle, lamellar and interlamellar epithelial exfoliation in *Salmo gairdneri* [56]. Upon intoxication with 10 ppm cationic surfactant of *Labeo rohita*, it was observed that the gill cells suffered distortions and degenerations [57]. The effects of cationic surfactants (used as biocide in fish diseases), which were studied on the gills of *Salmo gairdneri*, showed severe spongiotic lesions, necrotic lesions, lamellar fusion, membrane vesiculation, hydropic degeneration, exfoliation of lamellae and interlamellar epithelium [56].

The microscopic investigation of the structural changes in fish kidney exposed to BEC within our study showed abnormalities, such as an increase of melanomacrophages aggregation incidence, an expansion of Bowman space, a cytoplasm vacuolation of goblet cells lining the collecting tubules, and finally the loss of tubules epithelium integrity (Figure 6b–e).

Camargo and Martinez [58] identified in *Prochilodus lineatus* a glomerular capillary dilation, nuclear or cellular hypertrophy, cytoplasm vacuolation, tubular lumen dilation, tubular regeneration and melanomacrophage aggregation as abnormal changes within this tissue. Furthermore, a decreased Bowman space, the presence of blood in the Bowman space, a degeneration of hyaline droplets, tubular degeneration, a decreased tubular lumen caliber, are more severe and affect tissue function.

The histopathological consequences produced by the cationic surfactant BEC in fish tissues (gills and kidney) could also be explained by the fact that these organs present a good representation of polyunsaturated fatty acids needed to preserve membrane fluidity [51], but the phospholipid membranes are very sensitive to surfactants actions [25] or to their ROS products [59].

There are small chances that these toxic effects recorded in laboratory conditions in the case of *Cyprinus carpio* contaminated with BEC (with analytical concentration of 0.78 ± 0.15 mg/L) will also occur in the natural environment if we take into consideration the low concentrations detected in surface waters [10,11,12,13] obtained through rivers dilution and other biotic and abiotic factors that may cause biodegradation or absorption on certain substrates. However, a major risk results from the occurrence of recalcitrant metabolic compounds that can be released into surface water after the treatment of wastewater contaminated with cationic surfactants [14], about which we still have many knowledge gaps. Considering that there are no standard limits for these compounds in surface waters, while they have been used more and more, the low biodegradation, persistence and incidence of recalcitrant metabolites should be considered a threat for aquatic life.

Nevertheless, there are still several limitations in the research of toxic effects exerted by these compounds, such as: (i) gaps in the detection methods of the original compounds and metabolites; (ii) the influence of biotic and abiotic factors that may influence bioavailability and toxicity; (iii) the upgrading of current legislation for monitoring quaternary ammonium compounds in discharged wastewater.

## 5. Conclusions

Our study showed the sensibility of gills and kidneys of *Cyprinus carpio* (common carp) to an acute toxicity induced by a cationic surfactant (BEC) in terms of histological changes and oxidative stress induction. Besides the alteration of the antioxidant enzymes activities after 96 h of exposure, an increased lipid peroxidation and decreased GSH confirmed that BEC was able to induce toxicity to these tissues. The metabolic effects were correlated to the unspecific structural changes observed in gills and kidneys, having a moderate degree of severity and generally being compatible with life for the exposure time studied. Our research confirmed the utility of biochemical and histological analyses in the fish organs as tools to establish the water quality and ecotoxicological potential of chemicals.

## Data Availability

Data are available upon request to the corresponding author.
